# The role of extracellular matrix stiffness in regulating fibroblast behaviors and disease progression

**DOI:** 10.3389/fimmu.2026.1849447

**Published:** 2026-06-18

**Authors:** Zezhi Fan, Kaiyuan Deng, Lin Wang, Jiangjiang Fu, Li Ma, Shiwei Tu, Junyi Li, Kaifang Yao, Zhifang Xu, Yi Guo

**Affiliations:** 1 Research Center of Experimental Acupuncture Science, Tianjin University of Traditional Chinese Medicine, Tianjin, China; 2 Department of Traditional Chinese Medicine, Qinghai University Medical College, Xining, China; 3 National Clinical Research Center for Chinese Medicine, Tianjin, China; 4 Tianjin Key Laboratory of Modern Chinese Medicine Theory of Innovation and Application, Tianjin, China

**Keywords:** extracellular matrix stiffness, fibroblasts, fibrosis, mechanotransduction, tumors

## Abstract

As a representative physical factor in the extracellular matrix (ECM), ECM stiffness regulates fibroblast behaviors and contributes to physiological homeostasis and pathological processes. This process relies on mechanosensors, including integrin-based adhesion complexes, mechanosensitive ion channels, and discoidin domain receptors (DDRs), which detect changes in stiffness and transduce them into biochemical signals. Through downstream pathways including YAP/TAZ and RhoA/ROCK, ECM stiffness modulates essential cellular behaviors such as adhesion, migration, proliferation, and differentiation, adapting to the requirements of tissue homeostasis. Under pathological conditions, abnormal elevation of ECM stiffness occurs in tumor and fibrotic tissues, forming a vicious cycle of ECM deposition-increased stiffness-abnormal cellular activation. Therefore, deciphering the regulatory mechanisms of ECM stiffness on fibroblasts not only deepens the understanding of the interaction between cells and the mechanical microenvironment but also provides crucial theoretical support and innovative ideas for the targeted therapy of diseases such as tumors and fibrosis, as well as the development of tissue engineering and regenerative medicine.

## 1 Introduction

As a core component of the extracellular microenvironment for cell survival, the ECM is a complex network structure composed of macromolecular substances secreted by cells. The biochemical components of the ECM are mainly divided into two categories: interstitial matrix and basement membrane. The interstitial matrix is centered on type I/III collagen, fibronectin, elastin, and various proteoglycans, forming a loose collagen fiber network. The basement membrane, on the other hand, consists of type IV collagen, laminin, nidogen, and heparan sulfate proteoglycans, forming a dense sheet-like network that separates cells from the surrounding matrix (1, 2). The cross-linking and arrangement of these components endow the ECM with diverse geometric configurations and spatial morphologies, providing external support for cells and transmitting essential biochemical signals for function, playing a core role throughout the entire life cycle of organisms (3). In common immune responses such as inflammation, injury, infection, and aging processes, tissue remodeling caused by changes in the content of hyaluronic acid, collagen, proteoglycans, etc., produces ECM fragments that guide the migration and infiltration of immune cells such as T cells and neutrophils into inflamed tissues (4–6), inhibit Toll-like receptor signaling or activate immunosuppressive receptors, reduce the production of inflammatory factors, and simultaneously regulate cytoskeletal remodeling and downstream gene expression (7, 8), affecting intercellular signal transduction and communication networks, altering the morphology and function of organelles such as intracellular mitochondria, and providing additional defense capabilities (9).

With the deepening of life science research, simple biochemical signal regulation can no longer fully explain complex biological behaviors such as dynamic adaptation and functional choices of cells in the complex microenvironment. Many diseases, disorders of tissue and organ function are caused by changes or even abnormalities in the mechanical properties of biological tissues: In the development of common atherosclerosis, the proliferation and migration of vascular smooth muscle cells lead to a large amount of matrix collagen deposition, cross-linking of extracellular matrix proteins, and gradual increase of vascular wall stiffness (10). The abnormal mechanical microenvironment of cancer and fibrosis shows the imbalance of TGF-β (11). In inflammatory disease models, dendritic cells under high stress showed enhanced activation and increased flux of major glucose metabolic pathways (12). The key physical properties of the ECM include matrix stiffness, matrix topology, matrix viscoelasticity, matrix ductility, and matrix compliance (13, 14). These physical factors are highly tissue-specific. As an index used to measure the hardness and softness of a material, stiffness can be understood as the ability of a material to resist elastic deformation, shaping deformation or failure. However, from the perspective of cell culture, different culture materials will have an impact on cell growth, matrix hardness is different, the tensile stress cells feel is different, the transformation and development direction of cells are different, so the cell physiological activities are also different, which have an important impact on cell proliferation, adhesion, spreading, migration, differentiation and apoptosis. It is a complex process that transforms from mechanical pathway to biochemical pathway.

Closely related, fibroblasts are not only the core effector cells for ECM synthesis and remodeling, actively shaping and maintaining the tissue-specific ECM microenvironment by precisely regulating the synthesis and secretion of key components such as collagen, elastic fibers, glycosaminoglycans, and reticular fibers, but also the key carriers for mechanosignal perception and response (15–17). Their core biological behaviors such as adhesion, migration, proliferation, and differentiation are all precisely regulated by ECM stiffness. Under physiological conditions, fibroblasts can dynamically adjust their synthetic and secretory activities, participating in the continuous remodeling of the ECM, thereby maintaining the stability of tissue structure and function, and providing key support for tissue repair and reconstruction. In pathological states such as tumors and fibrosis with abnormal deposition of ECM components, fibroblasts are continuously activated, prompting them to overexpress ECM components, forming a vicious cycle of ECM deposition - increased stiffness - continuous cellular activation. Therefore, deciphering the regulatory mechanisms of ECM stiffness on fibroblasts not only deepens the understanding of the interaction between cells and the mechanical microenvironment but also provides a new perspective for cracking the pathological processes of diseases such as tumors and fibrosis.

## 2 ECM stiffness and fibroblasts

As the most representative key physical signal, ECM stiffness exhibits high tissue specificity and precise functional regulation (18). Under physiological conditions, soft tissues such as nerves and adipose tissue have low stiffness (0.1–1 kPa), while hard tissues such as muscle and bone have high stiffness (above 8 kPa) (19). Studies on the differentiation of bone marrow mesenchymal stem cells (MSCs) on hydrogel culture media with different stiffness have confirmed that under hard stiffness conditions of 39~45 kPa, the expression of osteogenesis-related differential genes increases, and cells tend to differentiate into osteoblasts. In a low stiffness environment of 7~10 kPa (20), the expression of adipogenesis and chondrogenesis-related genes such as WNT2B, MEF2C, COL2A2, and MMP9 is upregulated, and cells are prone to form lipid droplets and differentiate into adipocytes (21). Meanwhile, the stiff matrix can promote glycolysis and oxidative phosphorylation of MSCs, enhance the antioxidant defense system, thereby playing a role in osteogenic differentiation (22). In addition, macrophages can regulate their functions and behaviors by perceiving the matrix rigidity of the surrounding environment. When macrophages are in a matrix with high stiffness, they exhibit strong M2-type characteristics such as adhesion, migration, differentiation, and intracellular signal transduction. In a relatively soft matrix, macrophages present a nearly round shape with relatively low adhesion and migration capabilities, showing M1-type characteristics (23, 24). The intrinsic cause of ECM stiffness alterations lies in changes to its composition and structure. Collagen is the most abundant component of the ECM, which is categorized into fibrous collagens (including types I–III, V and XI) and non-fibrous collagens remodelling the extracellular matrix in development and disease (25).

Fibroblasts differentiate from mesenchymal cells during embryonic development, are core constituent cells of connective tissue, and are receptive and effector cells under mechanical force. When stimulated by external mechanical forces, they transform from a quiescent state to an activated state, producing high levels of ECM components such as collagen, elastic fibers, glycosaminoglycans, and reticular fibers, participating in the synthesis and remodeling of the extracellular matrix, and maintaining tissue structure and function. Meanwhile, matrix stiffness can also induce fibroblasts to change their morphology and affect their adhesion, migration, proliferation, differentiation, and other behaviors.

Fibroblasts need a rigid environment to form mature focal adhesion and activate actin stress fibers. Focal adhesion is the core structure, and its maturity directly determines the strength and stability of adhesion. An earlier study found a linear relationship between vinculin recruitment area in focal adhesion and residence time with increasing matrix stiffness (3 kpa, 5 kpa, and 14 kpa), whereas this relationship was disrupted on soft substrates (26). Fibroblasts cultured at 9 kpa for 96 hours were less mature and smaller than those cultured at 30 kpa, and could not form F-actin bundles (27). A study culturing fibroblasts in a stiffness range of 2~55 kPa found that 3 kPa may be a critical stiffness threshold for fibroblasts to exert adhesion (28). On soft matrices below 3 kPa, the cell spreading area is extremely small, adhesion ability is weak, and there are no obvious stable cell-matrix adhesion structures. Beyond this stiffness, the cell spreading area and integrin content increase suddenly and significantly, and adhesion stability is sharply enhanced. With the increase of ECM stiffness, fibroblasts actively recruit more integrins capable of withstanding mechanical forces to the interface in contact with the ECM, providing a stronger molecular basis for adhesion while enhancing the interaction frequency between integrins and the ECM, thereby driving the maturation of adhesion plaques and strengthening adhesion function (29). Specifically, a stiff matrix of 21.5 kPa promotes fibroblasts to form large-sized, uniformly oriented adhesion plaques and anisotropically arranged stress fibers. This structure can not only anchor cells and the ECM more stably, providing stronger adhesion support but also lay the foundation for subsequent morphological changes such as cell elongation through ordered arrangement. Moreover, cells initiate the polarization program only when the adhesion plaques on the stiff matrix complete ordered orientation (30). In contrast, a soft matrix of 2.5 kPa forms numerous small-sized adhesion plaques arranged radially and disorderly, paired with radial stress fibers, which cannot provide stable mechanical anchor points, resulting in weak cell adhesion strength and difficulty in forming regular adhesion-mechanical signal transmission (31).

The migration behavior of fibroblasts is closely related to the adhesion level and also inseparable from the key regulation of ECM stiffness (32), mainly reflected in two dimensions: migration speed and migration tropism. In terms of speed, the traditional view holds that fibroblasts tend to migrate efficiently on stiff matrices because cells form more mature and stable adhesion on stiff matrices (33, 34), while on soft matrices, cells are difficult to establish stable mechanical support points, and most adhesions retract and slip rapidly after formation, leading to the failure of the cell leading edge to advance effectively (35, 36). Interestingly, when fibroblasts were treated with TGF-β, their force on softer substrates was increased, suggesting that TGF-β enhanced the mechanical sensitivity of fibroblasts and increased the rate of migration even in a less rigid environment. In contrast, on rigid substrates, TGF-β treatment induces a shift to a myofibroblast phenotype, manifested as reduced migration velocity, and more focus on matrix remodeling, not through force generation to achieve migration (27). Recent studies have found that moderately inhibiting myosin contractility in fibroblasts on soft matrices can instead stabilize adhesions and accelerate cell migration on soft matrices, suggesting that there is a mechanical balance threshold for the adhesion between cells and the ECM during migration. Appropriately reducing the contractility of cells on soft matrices can reduce retraction and slip during post-adhesion migration (37). On the other hand, actin-myosin activity uses up a substantial fraction of intracellular energy, so rigid matrixes mediated mitochondrial division helps to transport mitochondria to lamellipodia and filopodia to provide energy for the cell to generate traction for migration (38). In terms of tropism, fibroblasts migrate irregularly in a uniform matrix environment, while in an environment with a mechanical gradient, the cell membrane and cytoskeleton rearrange, integrins bind to ECM ligands, promoting the phosphorylation and activation of adhesion plaques and their complexes, connecting to filamentous actin through adapter proteins such as talin, contracting and pulling actin, and enhancing the affinity between integrins and their substrates (39). This binding speed is faster on stiffer substrates, leading to the aggregation of more integrins, thereby increasing the generation of traction forces and making cells biased towards stiffer regions (40, 41). At the same time, the high-stiffness matrix induces fibroblasts to highly express α-smooth muscle actin (α-SMA), which assembles into stress fibers to enhance cell contractility, providing motivation for migration (42).

In terms of proliferation, the increase in extracellular matrix stiffness initiates intracellular DNA synthesis and cell division signals through a series of pathways such as driving cytoskeletal reorganization, intracellular protein transduction, and changes in specific gene expression in fibroblasts, accelerating cell proliferation. It is relatively clear that the nuclear translocation of YAP protein binds to the transcriptional coactivator TAZ to transcribe the enhancer factor TEAD and genes related to cell proliferation (43). This regulatory rule has been reflected in studies on fibroblasts from different tissue sources: in experiments related to lung fibroblasts, a stiff matrix of 50 kPa can reduce cell apoptosis and maintain stable cell numbers through activating the RasGRF1 signaling pathway closely related to cell proliferation and survival, laying the foundation for subsequent proliferation (44); in cardiac fibroblasts, cardiac fibroblasts on a stiff ECM of 50 kPa show enhanced proliferation compared to a soft ECM of 0.5 kPa, which is related to the enhanced activation of the transcriptional cofactor YAP increasing the transcriptional activity of TEAD and RUNX2 transcription factors (45); in breast fibroblasts, the increase in stiffness increases the cell spreading area and nuclear aspect ratio, making the cytoskeleton in an extended and active state, providing structural support for cell division (46); in studies on human dermal fibroblasts, increasing matrix stiffness *in situ* through a stiffness-tunable collagen scaffold activates the proliferation program of originally slow-proliferating cells, and their proliferation rate can be close to that of cells continuously cultured on stiff matrices (47), suggesting that soft matrices maintain cells in a quiescent state due to insufficient mechanical signals, and the proliferation potential of cells can be reactivated with the enhancement of stiffness signals.

Matrix stiffness is also a key mechanical signal regulating fibroblast differentiation and trans-lineage reprogramming. The differentiation trend of fibroblasts shows precise tissue matching with matrix stiffness: a matrix stiffness of 0.1–1 kPa tends to initiate their differentiation towards the neural direction, while stiffnesses of 8–17 kPa and 25–40 kPa promote their reprogramming towards the cardiac muscle and bone directions, respectively (48). At the mechanistic level, a soft matrix of 100 Pa simulates brain ECM stiffness. It was found that such a soft matrix can promote fibroblasts to break away from their original functional state and successfully reprogram towards neurons by inhibiting microRNA-615-3p, targeting and activating integrin β4, and regulating the downstream integrin-actin cytoskeleton and Hippo signaling pathway, ultimately forming glutamatergic neurons with electrophysiological functions, which cannot be achieved by traditional rigid stiff matrices (49). Increasing the matrix stiffness from 6 kpa to 36 kpa, or the addition of 5 to 10 ng/mL of TGF-β alone, resulted in increased SMA expression levels in both dermal and mouse fibroblasts, promoting the incorporation of smooth muscle actin into the actin cytoskeleton, and thus the transformation into myofibroblasts. It is suggested that besides biochemical factors, the change of environmental stiffness is another factor affecting the phenotype of fibroblasts (27). In contrast, in the muscle-related tissue microenvironment, a relatively stiff matrix of 23 kPa significantly increases the expression of α-SMA in fibroblasts, enhances cell contractility, and promotes their differentiation into myofibroblasts, while a soft matrix of 1.3 kPa mostly maintains the quiescent phenotype of fibroblasts. In addition, the physiological increase in local tissue stiffness can further promote the cell differentiation process (50). For example, in a stiff matrix formed by compression, the sensitivity of corneal keratinocytes to differentiate into fibroblasts is significantly enhanced: not only the strong profibrotic factor TGFβ1 can induce differentiation, but FGF, which is ineffective in soft matrices, can also trigger the formation of stress fibers and promote active cell differentiation, suggesting that stiff matrices can create a microenvironment more prone to initiation for cell differentiation by lowering the differentiation threshold and amplifying pro-transformation signals (50).

However, most of the above studies on the mechanism of force transmission between fibroblasts and ECM focus on the two-dimensional culture system, which is a simulation and simplification of the *in vivo* microenvironment. However, *in vivo* cells usually interact with the extracellular matrix in a three-dimensional (3D) environment. Compared with traditional two-dimensional methods, 3D culture also increases the microstructure, porosity and elastic behavior of the extracellular matrix. Induced upregulation of the expression of genes encoding different types of collagen, fibronectin, laminin, etc (51). The interaction between cells and ECM in 3D environment may be partially different from that in 2D environment: in 2D environment, cells can spread freely on the substrate, while in 3D environment, cell volume and shape changes will be physically resisted or limited by the surrounding ECM. On a two-dimensional matrix, cells form adhesion to extracellular matrix on only one surface. A matrix with sufficient rigidity and ligand density usually induces focal adhesion. In the three-dimensional environment, cells can form adhesion in all directions, and the mechanical efficiency and coupling between multiple adhesion structures and the local extracellular matrix structure jointly regulate the stability of adhesion (52). At the same time, the increased porosity of collagen gel promotes cell migration more rapidly and reduces the need for proteolysis of extracellular matrix. Studies have shown that if the pore size of the extracellular matrix is larger than about 3 μm, cells can efficiently migrate by squeezing through these pores. Excessive matrix thickening and reduced porosity, although increasing ECM stiffness, will inhibit normal fibroblast migration, while oriented surface alignment can enhance cell migration (37, 53, 54). In addition, either the increased rigidity or the enhanced degradability led to the aggravation of nuclear folding in 3D conditions (55). However, in 2D conditions, increased rigidity will have the opposite effect on nuclear folding, and it is likely that the nuclear force conduction mechanism in 3D will be different from that in 2D.

In summary, as core cells of connective tissue, fibroblasts have both mechanical force sensing and effector functions, and their adhesion, migration, proliferation, and differentiation behaviors are all precisely regulated by ECM stiffness. In terms of adhesion, 3 kPa may serve as a critical threshold. On soft matrices below this stiffness, the cell spreading area is small and adhesion structures are unstable. Beyond this threshold, integrin recruitment increases, and stiff matrices are more likely to form large-sized, ordered adhesion plaques, laying the foundation for cell morphological changes. In contrast, soft matrices only form scattered and disordered adhesion structures with weak mechanical anchoring effects. In terms of migration, stiff matrices support efficient cell migration through stable adhesion, while moderately inhibiting myosin contractility in soft matrices can stabilize adhesion and accelerate migration to a certain extent. Moreover, cells have the characteristic of tropic migration towards high-stiffness regions, obtaining migration motivation through integrin aggregation and enhanced contractility. At the proliferation level, stiff matrices activate DNA synthesis and cell division signals by driving cytoskeletal reorganization and activating pathways such as YAP/TAZ and RasGRF1, awakening cell proliferation potential, while soft matrices maintain cells in a quiescent state. In terms of differentiation, stiffness regulation shows clear tissue matching: 0.1–1 kPa promotes neural differentiation, 8–17 kPa is conducive to cardiac muscle reprogramming, and 25–40 kPa promotes bone differentiation. Furthermore, stiff matrices can lower the differentiation threshold and amplify pro-transformation signals, creating a microenvironment more prone to initiation for cell differentiation. However, due to insufficient surface topography, surface stiffness, cell-cell interactions, and interactions between cells and extracellular matrix, 2D cell culture systems cannot fully mimic the cell physiological state observed in healthy tissues *in vivo*. Given these shortcomings, there is an urgent need to develop novel and adaptive cell culture systems. Three-dimensional super-resolution imaging, molecular force sensors, and materials with dynamically tunable mechanical properties are emerging technologies that promise to meet this need, providing detailed readings of the dynamic molecular-scale interactions and forces occurring between cells and substrates. This helps to form a more comprehensive understanding of cell-matrix signaling under different stiffnes.

### 2.1 Cellular mechanosensors

#### 2.1.1 Integrin adhesion plaque complexes

Integrins are transmembrane receptors composed of α/β subunits (56). Their extracellular ends can bind to ligands such as collagen and fibronectin in the extracellular matrix, and their intracellular ends are connected to the cytoskeleton through adapter proteins, serving as a bidirectional transduction hub for mechanical and biochemical signals (57, 58). When cells contact the matrix, integrins are activated and aggregated, recruiting force transmission-related proteins (talin, vinculin, paxillin), focal adhesion kinase (FAK, c-Src), and other molecules to form adhesion plaques (59, 60). ECM stiffness directly determines the state of adhesion plaques: large, mature, and stable adhesion plaques are formed on stiff matrices, while only loose focal adhesions are formed on soft matrices (29, 61). Proteomic data of multiple sets of integrin adhesion plaque complexes show that the expression and activation of adhesion proteins are stiffness-dependent. In fibroblasts cultured on stiff matrices, the expression and phosphorylation levels of common adhesion proteins talin, vinculin, FAK, and c-Src are significantly higher than those in the soft matrix group (62–64).

In the soft matrix group, common adhesion proteins have basic expression but are mostly in an inactive state, and their binding efficiency to the cytoskeleton is low, unable to effectively transmit stiffness signals. The maturity of adhesion plaques affects the adhesion stability and migration ability of fibroblasts on matrices with different stiffnesses. Mature adhesion plaques promote the transformation of fibroblasts into contractile myofibroblasts and initiate the long-term proliferation and migration of fibroblasts (65). In addition, ECM stiffness can regulate the perception and transmission efficiency of mechanical forces by fibroblasts by changing the recruitment ratio of talin, vinculin, and paxillin in the integrin adhesion plaque complex: under a stiff matrix stiffness of 47 kPa, the complex forms a high-efficiency force transmission type with a low vinculin ratio, and the protein stoichiometric ratio is talin:vinculin:paxillin=1:2:2 at a traction force of 60 pN. This ratio is more suitable for the precise perception of matrix sclerosis in the early stage of physiological wound healing. In contrast, in the soft matrix of 12 kPa, the complex forms a force-buffering type with a high vinculin ratio, and the ratio changes to 1:6:3 under the same traction force, adapting to the low-sensitivity perception of the quiescent state of normal skin soft tissues (62). This adaptation mechanism enables fibroblasts to maintain basic mechanical perception ability in microenvironments with different stiffnesses and exert corresponding physiological functions. The other end of the connection between adhesion plaques and the intracellular actin cytoskeleton can promote actin assembly by regulating RhoA signals, forming contractile stress fibers, which regulate force transmission like a clutch. Under stiff matrices, the clutch is tightly combined, and force transmission efficiency is high (59), which can not only support cytoskeletal contraction but also inhibit the phosphorylation of YAP/TAZ proteins in the Hippo pathway, allowing dephosphorylated YAP/TAZ to dissociate from the cytoplasmic inhibitory complex, enter the nucleus, and bind to TEAD transcription factors, thereby initiating the transcription of Col I and α-SMA related to proliferation, survival, migration, and ECM synthesis (56, 66). Under soft matrices, the “clutch” is less activated, and force transmission is weak, unable to block the Hippo pathway, leading to continuous phosphorylation of YAP/TAZ and retention in the cytoplasm, unable to exert transcriptional regulation, and cells thus maintain a quiescent phenotype with significantly reduced functional activity.

#### 2.1.2 Mechanosensitive ion channels

In addition to integrin-mediated signaling pathways, mechanosensitive ion channels also play a key role in the mechanotransduction of extracellular matrix stiffness. With the increase of ECM stiffness, the opening frequency of Piezo1 channels increases exponentially (67). In this process, changes in stiffness can induce conformational changes in the extracellular “cap”-like domain and three peripheral mechanosensitive “paddles” of Piezo proteins, thereby promoting the opening of the central pore of the channel. Transient Ca²^+^ influx initiates downstream signal transduction (68). Activated Piezo1 further activates the integrin-FAK signaling pathway and triggers the nuclear localization of YAP, thereby synergistically promoting the proliferation, differentiation, and migration of fibroblasts. A study on human fibroblasts showed that when cells are cultured on a stiff gel of 50 kPa, the expression of Piezo1 protein reaches 2.1 times that of the soft gel group of 2 kPa, while knocking down Piezo1 with siRNA prevents the stiff ECM from inducing the upregulation of Piezo1 expression. In-depth studies have found that the core indicators of fibroblast activation are significantly enhanced on a stiff ECM of 50 kPa: the proportion of α-SMA-positive cells is 2.3 times that of the soft ECM, and the expression levels of collagen and fibronectin are increased by 1.8-2.0 times. After knocking down Piezo1, these activation indicators return to the level of the soft ECM group, suggesting that Piezo1 is an essential switch for stiff ECM to induce fibroblasts to transform into a profibrotic phenotype (69). In addition, studies on fibroblasts from different tissue sources have found that compared to human atrial fibroblasts cultured on a soft gel of 3 kPa, activated Piezo1 in those cultured on a stiff gel of 6 kPa can not only drive actin cytoskeleton reorganization through FAK but also affect surrounding cells not expressing Piezo1 by secreting the cytokine interleukin-6, realizing the coordinated stiffness adaptation at the population level of fibroblasts (70). After remodeling the ECM stiffness around senescent skin fibroblasts with poly-L-lactic acid fillers (71), the ERK1/2-AKT pathway downstream of Piezo1 and mTOR and its downstream effectors can be activated respectively, providing support for tissue regeneration by upregulating cell cycle regulatory proteins and enhancing collagen synthesis in senescent fibroblasts, providing evidence that Piezo’s regulation in response to stiffness is tissue-specific.

Multiple members of the transient receptor potential (TRP) ion channel family also have mechanosensitivity and participate in various sensory processes through signaling lipids and physical stimuli. Among them, TRPV4, as a non-selective calcium-permeable cation channel, can respond to osmotic, thermal, and mechanical stimuli, and is another widely recognized ECM “stethoscope” after Piezo1 (72). When ECM stiffness changes, TRPV4 is activated and affects extracellular Ca²^+^ influx (73). On the one hand, it activates the RhoA/ROCK signaling pathway and the reorganization and dynamic changes of the cytoskeleton, realizing the conversion of mechanical signals into changes in cell morphology and function (74). On the other hand, it activates intracellular PI3K-AKT, PKC, and other signaling pathways related to collagen synthesis, promotes the expression of collagen synthesis-related genes COL1A1 and COL3A1 to increase the production of new collagen fibers, and regulates the activity or expression of matrix metalloproteinases to participate in the collagen degradation process (75). It not only avoids excessive collagen deposition leading to ECM stiffness but also prevents excessive degradation causing ECM damage, maintains the balance of collagen synthesis, remodeling, and degradation, and ensures that ECM stiffness is stable within the physiological range. In addition, during the YAP/TAZ nuclear translocation required for cell transformation, the increase in ECM stiffness induces the activation of TRPV4, promoting YAP nuclear shuttling and downstream signal transduction. Antagonizing TRPV4 leads to the accumulation of the YAP inhibitory protein AMOT in the cytoplasm, which continuously anchors YAP/TAZ, preventing its nuclear shuttling and weakening the synergistic activation of Smad2/3 and YAP (76). Another study cultured human skin fibroblasts with 0.5 kPa, 8 kPa, and 25 kPa, and found that the higher the matrix stiffness, the higher the expression of TRPV4, which integrates matrix stiffness mechanical signals with TGF-β soluble signals, enhancing TGF-β-induced cell differentiation. In turn, TGF-β promotes the expression of TRPV4, forming a closed loop to jointly promote transformation. At the same time, the transduction of the TGF-β1/non-Smad pathway to induce fibroblasts to differentiate on stiff matrices also requires the participation of TRPV4, suggesting that TRPV4 is indispensable in the process of fibroblast transformation and differentiation (77, 78).

#### 2.1.3 DDRs

As emerging mechanosensors for stiffness perception, DDRs belong to tyrosine kinase receptors, including two subtypes: DDR1 and DDR2 (79). DDR1 was first discovered in adipose-derived stem cells as a mechanical sensing element that promotes the expression of aromatase on softer substrates. Subsequently, in fibroblasts, the phosphorylation level of DDR1 increases with the increase of substrate stiffness, and this process depends on type I collagen ligands. Fibroblasts not only secrete collagen themselves but also interact with ECM collagen through collagen receptors such as DDRs and integrins, secondarily regulating their own functions and extracellular matrix remodeling (80). The deletion of DDR2 weakens the response of lung fibroblasts to collagen fiber stiffness, reduces β1 integrin activation, focal adhesion formation, and entanglement between dendritic protrusions and collagen, thereby inhibiting cell spreading, lamellipodia formation, and migration (81, 82). After collagen fibers activate DDR2, they can also participate in the synthesis, secretion, and degradation of collagen by regulating matrix metalloproteinases. The activation mechanism of DDR1 is unique: it exists as a dimer in the inactive state, and undergoes “liquid-liquid” phase separation (LLPS) under the stimulation of extracellular matrix stiffness and collagen, and phase separates with LATS1 to inhibit the activation of the Hippo pathway. At the same time, it can bind to myosin IIA activated by MLCK, optimizing the transmission of contractile force to collagen, and promoting cell contraction and collagen rearrangement (83). Unlike the transient phosphorylation of general tyrosine kinase receptors, DDR1 can undergo sustained phosphorylation for up to 16 hours after being activated with collagen as a ligand (84). Meanwhile, the activation of DDR1 may be secondary to integrins and mechanical ion channels, and jointly transmit collagen binding signals to the cytoskeleton, participating in the occurrence and development of various cell behaviors (85, 86).

At the same time, these receptors cooperate with each other under the influence of stiffness: multiple experiments have observed a cross-communication effect between DDR1 and integrins (87); the activation of Piezo1 interacted with TRPV4 to trigger a sustained Ca²^+^ signal, leading to mitochondrial depolarization, inhibition of PLA2 reduced the amplitude and duration of Piezo1-induced Ca²^+^ response through TRPV4 (88); under pathological conditions, Piezo1 stimulation triggered TRPV4 channel opening, which was responsible for the sustained elevation in intracellular calcium that caused intracellular organelle dysfunction (87); DDR1 associates with TRPV4 in cell-matrix adhesions to enable calcium-regulated myosin activity and collagen compaction (86).

### 2.2 Intracellular mechanotransduction pathways

Stiffness information affects intracellular signal transduction through different molecular pathways and enters the nucleus to regulate gene expression. As the center of transcription, YAP/TAZ and RhoA/ROCK are key elements of mechanotransduction pathways downstream of integrins and mechanosensitive ion channels.

#### 2.2.1 YAP/TAZ pathway

YAP/TAZ are readers of various mechanical signals, integrating multiple upstream signaling pathways into the nucleus (89). The Hippo signaling pathway, as the classic upstream of YAP/TAZ, starts with MST1/2 and LATS1/2, directly regulating the phosphorylation state of YAP/TAZ. Under normal conditions, the Hippo signaling pathway is active, and Yap is located in the cytoplasm in a phosphorylated form. After the increase of matrix stiffness, the activation of the Hippo pathway is inhibited, and Yap is activated and dephosphorylated, entering the nucleus, combining with nuclear transcriptional regulators (TEAD) to form a complex for transcriptional expression, and promoting the transcription of target genes by regulating connective tissue growth factor (CTGF) (45, 90, 91). The deletion of LATS1/2 can reduce the cytoplasmic localization of YAP induced by soft matrices (92). Inhibiting the destruction of Rho or F-actin blocks the transcriptional activity of YAP/TAZ (93, 94), while promoting F-actin polymerization and stress fiber formation enhances its activity, revealing that actin transmits upstream stiffness changes to the Hippo-YAP axis, converting changes in the physical microenvironment into regulation of gene transcription (95). At the same time, as a signal hub, YAP/TAZ can also crosstalk with multiple classic pathways such as Wnt, TGFβ, and Notch, integrating mechanical signals and biochemical signals to regulate cell fate determination.

#### 2.2.2 RhoA/ROCK pathway

RhoGTPases belong to small GTPases of the Ras superfamily, are major participants in cytoskeleton dynamics (96), and play a key regulatory role in cytoskeleton reorganization and subsequent changes in cell behaviors in response to changes in the mechanical environment (97, 98). Among them, RhoA is an important member of the Rho GTPase family, and the RhoA-ROCK signaling pathway is usually located at the junction between multiple mechanosensors and the cytoskeleton.In response to matrix stiffness, focal adhesion kinase (FAK) is phosphorylated and activated, recruiting paxillin and vinculin to form mature focal adhesions. The RhoA-ROCK pathway enhances cell contraction by phosphorylating myosin light chain (MLC), making focal adhesions generate dynamic traction tension, guiding fibroblasts to migrate towards high-stiffness regions (99, 100). Conversely, mature integrin focal adhesions directly bind to and activate Rho guanine nucleotide exchange factor (GEF) through FAK, promoting the conversion of RhoA from the GDP-bound inactive state to the GTP-bound active state (101). Other studies have shown that matrix sclerosis-induced upregulation of RhoA/ROCK activity in lung fibroblasts causes MKL1 to dissociate from G-actin and translocalize to the nucleus to interact with serum response factors to drive gene transcription. Co-immunoprecipitation analysis showed that MKL1 interacted with YAP and co-bound to the CCN1 (Cyr61) promoter. Knockout of MKL1 or YAP blocked the mechanical stretching-induced spreading and proliferation of primary mouse embryonic fibroblasts on soft substrates (102, 103). In addition, even if fibroblasts lack myosin contractility, they can regulate the viscoelastic properties of the actin network mediated by ARP2/3 and formin through the RhoA-ROCK pathway to achieve stiffness perception, optimize the efficiency of ion channel-mediated mechanical signal transmission, and ultimately promote YAP nuclear import or directly phosphorylate LATS1/2, inhibiting its phosphorylation of YAP, forming a core regulatory network together with YAP/TAZ (104), suggested that YAP/TAZ together with RhoA/ROCK exhibit complex interactions at multiple levels, which may contribute to the fine regulation of mechanical signaling and thus the regulation of gene transcriptional programs.

In summary, the YAP/TAZ and RhoA-ROCK pathways on fibroblasts, as core hubs throughout mechanical perception and signal transduction, extend from classic Hippo pathway regulation to multi-pathway crosstalk and cytoskeleton-nuclear membrane coordination. Through upstream and downstream synergistic effects with ion channels, integrin focal adhesions, and DDRs, they play important roles in perceiving changes in ECM stiffness and regulating differentiation, polarization, and cell transformation, constructing a multi-signal network for fibroblast stiffness perception.

## 3 Regulatory role of fibroblast response to changes in extracellular matrix stiffness in pathological processes

Under normal conditions, fibroblasts are responsible for producing and maintaining the extracellular matrix. When subjected to injury or other stimuli, they can transform into myofibroblasts, participating in inflammatory responses and rapid tissue repair. The formation of myofibroblasts is an emergency response to tissue injury, which stabilizes and repairs the injured area by producing a large amount of collagen. However, if this state persists for too long, it will lead to abnormal cross-linking and deposition of collagen, enrichment of fibronectin and glycoprotein components, combined with downregulated matrix metalloproteinase activity, collectively resulting in excessive ECM accumulation and metabolic imbalance, providing a key mechanical microenvironment basis for organ fibrosis and solid tumor progression. Fibroblasts play dual roles as “drivers” and “intervention targets” in this process.

### 3.1 Regulatory role of fibroblast response to changes in extracellular matrix stiffness in fibrosis

Fibrosis, as the terminal stage of disease-related tissue injury repair, is characterized by dysregulated tissue repair, manifested as excessive deposition of extracellular matrix, insufficient absorption, and increased stiffness. Meanwhile, activated myofibroblasts secrete more collagen and promote its cross-linking, further increasing matrix stiffness, forming a fibrotic vicious cycle of increased stiffness - cellular activation - further increased stiffness (105): the mechanically gated cation channel Piezo1 directly opens in response to increased stiffness, activates the Wnt2/Wnt11 pathway, and secretes the chemokine CCL24, which not only recruits immune cells but also accelerates the differentiation of fibroblasts into myofibroblasts, exacerbating collagen deposition. Conversely, the stiff ECM increases the expression of Piezo1, thereby enhancing the invasiveness of skin fibrosis. Integrins perceive stiffness by binding to collagen and fibronectin in the ECM, aggregate to form focal adhesions, and activate the downstream PI3K/AKT pathway, inhibiting fibroblast apoptosis and promoting fibrosis progression. In terms of the core link, the increased tissue stiffness drives the nuclear localization of YAP/TAZ (69). Compared to fibroblasts with conditional activation of YAP/TAZ on physiological matrices, the activation of YAP/TAZ on the stiff cellular matrix of fibrosis can overcome growth restrictions, accelerate the production of TGF-β (106), a universally recognized strongest profibrotic factor in tissues, amplify the profibrotic effect, upregulate the expression of α-SMA and type I collagen, enhance cell contraction and ECM secretion capacity, and ultimately maintain the pathological cycle continuously, making fibrosis difficult to reverse. Therefore, therapeutic strategies targeting fibroblasts can target the regulatory effect of ECM stiffness on the YAP signaling pathway to improve or reverse fibrosis. As existing studies have found, physiological stiffness (~1 kPa) can reverse the excessive activation phenotype of fibroblasts in idiopathic pulmonary fibrosis. Conventionally stiff silicones (2 MPa) coated with a soft silicone layer (2 kPa) reduced collagen deposition as well as myofibroblast activation,reduce reduce fibrosis induced by stiff silicone prostheses in mice (107). One is to target upstream molecules of the YAP signaling pathway for targeted intervention, such as inhibiting core kinase activity or dissolving ECM collagen, such as targeting matrix metalloproteinases to reduce matrix collagen cross-linking, targeting the mechanosensor SRC kinase to deactivate cardiac fibroblasts, reducing ECM rigid components, inhibiting lysyl oxidase-like protein 2 to reduce ECM stiff deposition metabolites (108, 109), using collagen-binding molecule-mediated drugs (such as pirfenidone) to carry collagenase to target fibrotic foci to improve matrix permeability (110), upregulate the expression of collagen metabolism-related proteins such as SEL1L (111), and enhance the endocytic degradation of excessive collagen by fibroblasts, thereby directly reducing matrix stiffness to regulate the abnormal activation of the YAP signaling pathway. The second is to target the activity of YAP/TAZ downstream of the Hippo/YAP signaling pathway and the interaction between YAP/TAZ-TEAD, breaking the pathological vicious cycle. For example, by knocking down or directly inhibiting YAP and TAZ, blocking the phosphorylation of Smad2/3 (91, 112–114), reducing the differentiation of fibroblasts into myofibroblasts, decreasing the synthesis of ECM components such as collagen I and fibronectin, indirectly regulating stiffness, and promoting fibrosis regression to achieve precise antifibrosis.

### 3.2 Regulatory role of fibroblast response to changes in extracellular matrix stiffness in tumors

In the past, tumor research has focused more on the biological mechanisms of tumor cells themselves. In recent years, the interaction between the extracellular matrix in the tumor microenvironment and cancer-associated fibroblasts (CAFs) has become a key perspective for deciphering tumor progression. CAFs remodel the tumor mechanical microenvironment by regulating the production and properties of ECM components such as collagen and hyaluronic acid, combined with increased solid stress and interstitial fluid pressure caused by tumor growth (115). Many solid tumors are found to be stiffer than surrounding tissues. The increase in ECM stiffness is positively correlated with malignant phenotypes such as tumor cell proliferation and invasion, which has been verified in various tumors: the stiffness of normal breast tissue is about 150 Pa, while that of breast cancer is 4 kPa. The stiff matrix triggers the production of the oncogenic actin regulatory protein MENA, which is known to participate in the formation of invadopodia, can degrade the ECM, and promote the unidirectional invasion of cancer cells into blood vessels for intravascular infiltration (115, 116). Cervical cancer cells are spherical with almost no migratory pseudopodia on a soft substrate of 1 kPa, while they are rich in pseudopodia on a stiff substrate of 20 kPa, and their migration ability is significantly enhanced (117). Hepatocellular carcinoma cells (Huh7, HepG2) are small and round on a 1 kPa substrate, while they spread and flatten on a 12 kPa stiff substrate, and their proliferation activity is significantly improved (118). The average area of ovarian cancer cells (SKOV3) on 3 kPa, 25 kPa, and glass substrates increases with the increase of stiffness, and their proliferation activity is synchronously enhanced (119). Studies on key signaling pathways have found that stiff matrices can activate AKT through the oncogene ZNF217 (120), thereby initiating the PI3K/Rac and ERK pathways. C-X-C chemokine receptor 4, as a key molecule in stiffness signal transduction, regulates the growth of hepatocellular carcinoma cells through the YAP pathway, regulates processes such as the cell cycle, DNA replication, and repair, directly stimulates tumor cell proliferation, and ultimately promotes malignant behaviors such as proliferation, invasion, and metastasis of hepatocellular carcinoma (121). In response to the above manifestations of abnormal extracellular matrix stiffness mediated by cancer-associated fibroblasts, therapeutic strategies focus on regulating the functional phenotype of cancer-associated fibroblasts and targeting stiffness remodeling (122), inhibitors targeting Hsp47, LOX, LOXL2, LOXL3, integrin, Piezo1, TRPV4, ILK, YAP/TAZ, and TEAD have also been developed. Many of these inhibitors have shown anticancer activity in preclinical studies. Specifically, the synthetic retinoic acid Am80 is used to induce CAFs to highly express the tumor suppressor marker Meflin protein, converting them from a protumor phenotype to an antitumor phenotype, and inhibiting collagen cross-linking activity by binding to lysyl oxidase (LOX), reducing abnormal ECM stiffening to lower tumor stiffness, and repairing tumor vascular function to improve the penetration efficiency of chemotherapeutic drugs (123). The indole-based fluoroallylamine PXS-51020A and PXS-5153A are LOXL2/LOXL3 inhibitors have antifibrotic activity in preclinical models of liver and lung metastasi. In addition, preclinical studies have demonstrated that PAT-1251/GB2064, a highly selective LOXL2 inhibitor based on a benzylamine with 2-substituted pyridine-4-ylmethanamines, has collagen accumulation-lowering and tumor-suppressing effect (124, 125). In addition to LOX, members of the matrix metalloproteinase (MMP) family are also core molecules through which CAFs regulate the synthesis and cross-linking of the ECM. Studies have demonstrated that batimastat, a broad-spectrum inhibitor targeting MMPs, can significantly suppress the secretion of MMP-2 and MMP-9 by CAFs in lung cancer tissues. This inhibition effectively reduces the abnormal deposition and cross-linking of collagen fibers, decreases the stiffness of tumor tissues, and thereby impairs the invasive and migratory capacities of tumor cells (126). TRPV4 antagonists have been developed in recent yea. Among them, GSK2798745 is the first TRPV4 blocker that has been evaluated in clinical trial. Early phase clinical trial has demonstrated that GSK2798745 is safe and well tolerated in human (127). In the context of combining immunotherapy with ECM stiffness remodeling, inhibition of ECM stiffening disrupts the tumor immunosuppressive microenvironment, leading to reduced interstitial fluid pressure and increased vascular permeability in tumor tissues. These biophysical changes further promote the maturation and antigen presentation of dendritic cells, enhance the activation of CD8^+^ T cells by PD-1 inhibitors, and ultimately result in a marked improvement in tumor inhibition rates in both melanoma and colorectal cancer models (128). Platelet-derived growth factor (PDGF) signaling can induce ordinary CAFs to transform into stiffness-induced CAFs, which construct an immunosuppressive barrier by inhibiting the activation and proliferation of CD8^+^ T cells to promote tumor escape. Injection of PDGF-neutralizing antibodies can reduce the number of stiffness-induced CAFs and restore the phenotype of PDGFRα^+^ antitumor CAFs, breaking immunosuppression and improving the infiltration and activation level of CD8^+^ T cells (129). Some of these potential therapeutic avenues have been translated into clinical trials. While the results of integrins inhibitors in clinical trials are largely disappointing, we still expect that encouraging results may emerge from other pipelines such as the Hippo/YAP pathway inhibitors. Of note, there may be many obstacles and challenges for targeting ECM stiffness in cancer, due to the complex roles of ECM in cancer progression and the dynamic nature of ECM remodeling.

**Figure 1:**
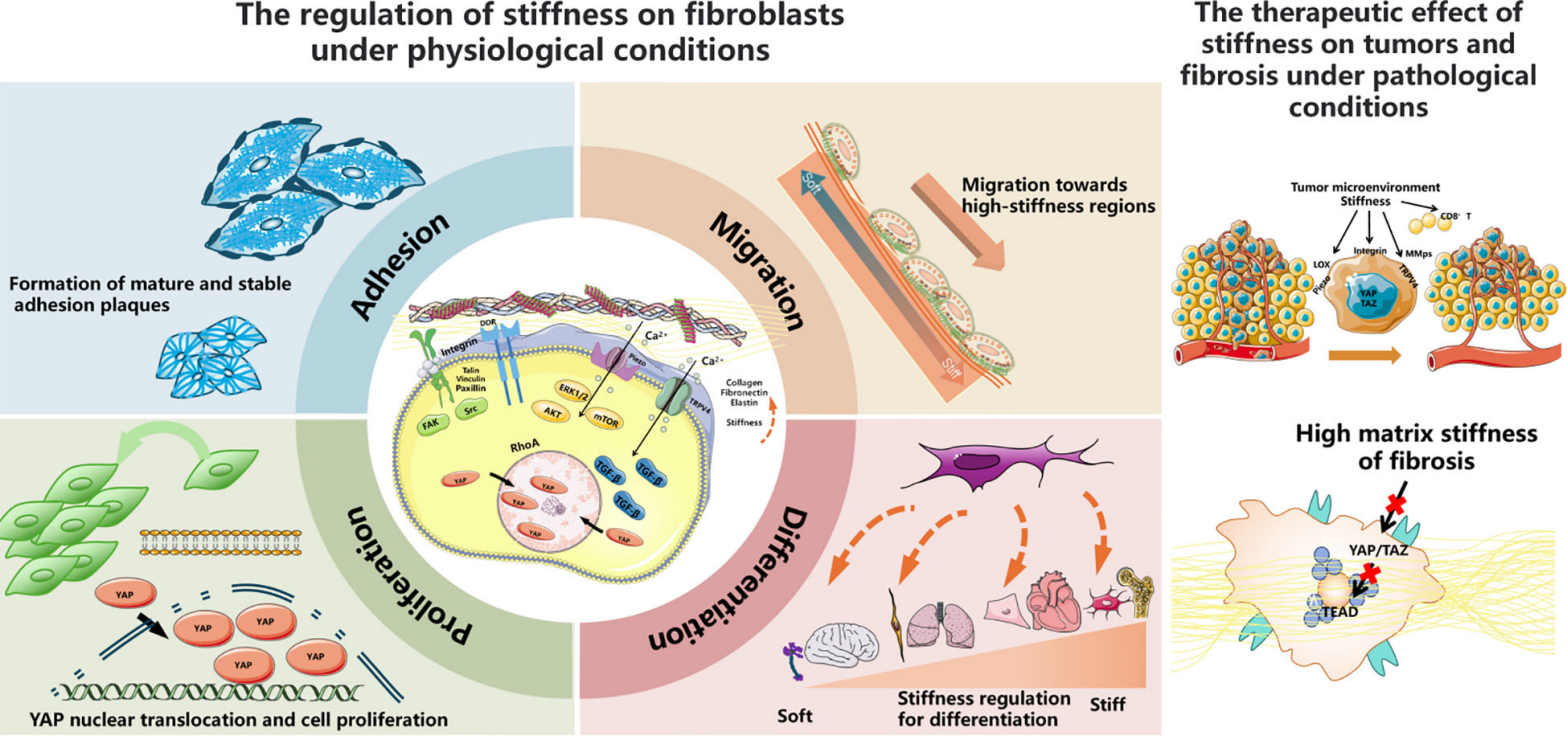
The stiffness of the extracellular matrix plays a regulatory role in physiology and a therapeutic role in pathology.

In summary, the continuous abnormal activation of fibroblasts leads to excessive ECM deposition and increased stiffness, making them key drivers and important intervention targets for organ fibrosis and tumor progression. In fibrosis, the increase in ECM stiffness activates YAP/TAZ through pathways such as Piezo1 and integrins, forming a vicious cycle. Treatments can target reducing matrix stiffness or directly intervene in the YAP pathway. In tumors, CAFs remodel the ECM to increase tumor tissue stiffness, promoting tumor cell proliferation and invasion. Treatments focus on regulating the CAF phenotype, inhibiting ECM stiffening, and improving the pathological microenvironment (**Figure 1**).

## 4 Discussion

As a core parameter of the cellular mechanical microenvironment, ECM stiffness serves as a pivotal bridge linking extracellular physical signals to intracellular biochemical reactions. The regulatory mechanisms underlying how ECM stiffness modulates fibroblast behaviors, together with their physiological and pathological significance, stand as a key research focus in the field of mechanobiology. Fibroblasts, as widely distributed stromal cells in the organism, function dual roles: they are not only the core effector cells responsible for ECM synthesis and remodeling but also critical carriers for mechanosignal perception and response. Their fundamental biological behaviors, including adhesion, migration, proliferation, and differentiation, are precisely regulated by ECM stiffness. For cell adhesion, 3 kPa represents a critical threshold: stiff matrices induce cells to recruit integrins, forming large-sized and orderly adhesion plaques that enhance adhesion stability, whereas soft matrices result in scattered and disorganized adhesion structures with weak anchoring capacity. In terms of migration, stiff matrices support efficient cell migration through stable adhesion; moderately inhibiting myosin contractility in soft matrices can accelerate migration. Additionally, cells exhibit mechanotaxis (a preference for stiffer environments), where high stiffness promotes migration toward stiffer regions by enhancing cellular contractility and traction forces. At the proliferation level, stiff matrices activate pathways such as YAP/TAZ and RasGRF1 to initiate cell division, while soft matrices maintain cells in a quiescent state. During differentiation, stiffness matches tissue-specific characteristics, enabling the precise reprogramming of fibroblasts into lineages such as neural cells, cardiomyocytes, and osteoblasts.

Fibroblasts perceive changes in ECM stiffness primarily through three classes of mechanosensors. Firstly, integrin adhesion plaques act as core mechanosensors. Through their α/β subunits, they bind to ligands such as collagen and fibronectin in the ECM, with their intracellular ends connecting to the cytoskeleton. On stiff matrices, integrins are extensively recruited and aggregated, recruiting proteins including talin and vinculin to form large, mature adhesion plaques with high force transmission efficiency. In contrast, soft matrices only induce loose focal adhesions with low levels of protein activation. By regulating the maturity of adhesion plaques and the recruitment ratio of associated proteins, integrin complexes adapt to the sensory demands of microenvironments with varying stiffness and activate downstream signaling pathways. Secondly, mechanosensitive ion channels, particularly Piezo channels and TRPV4, function in an interconnected manner. As matrix stiffness changes, the opening probability of these channels increases, leading to an exponential influx of Ca²^+^. This activates pathways such as integrin-FAK, RhoA/ROCK, and PI3K-AKT, promotes YAP nuclear localization, and participates in cytoskeletal reorganization and the maintenance of collagen metabolic balance. Thirdly, DDRs are emerging mechanosensors. With collagen as their ligand, DDRs can be activated downstream of integrins and other sensors, participating in mechanical perception in a coordinated manner.

Signals captured by these sensors ultimately form a mechanical-biochemical cross-amplification network via downstream YAP/TAZ and RhoA/ROCK pathways. This network drives adhesion plaque aggregation, actin cytoskeleton remodeling, and enhanced cellular contractility, providing motivation for cellular behaviors while reversely regulating upstream sensory efficiency to form a closed-loop regulatory system. (**Table 1**) Under pathological conditions, fibroblasts are overactivated by stimuli, leading to excessive synthesis and deposition of ECM components such as collagen and inhibition of ECM degradation. This results in abnormal ECM accumulation and increased matrix stiffness, forming a malignant cycle of “ECM deposition—increased stiffness—sustained cellular activation.” In fibrosis, excessive ECM deposition activates pathways including Piezo1 and integrins, promoting the differentiation of fibroblasts into myofibroblasts and exacerbating collagen deposition. In the tumor microenvironment, cancer-associated fibroblasts (CAFs) remodel the ECM to increase stiffness, which promotes tumor cell proliferation and invasion—with stiff matrices showing a positive correlation with malignant tumor phenotypes. Targeted interventions against this mechanism can be categorized into three strategies: (1) Regulating mechanosensors, such as inhibiting Piezo1 channels or targeting integrin/DDR receptors to block stiffness signal perception; (2) Interfering with downstream pathways, such as suppressing core pathways including YAP/TAZ-TEAD and RhoA/ROCK to prevent abnormal cellular activation; (3) Remodeling ECM stiffness, such as using collagenases or LOX inhibitors to reduce collagen cross-linking and deposition, or improving matrix permeability via targeted drugs, thereby providing novel strategies for fibrosis reversal and tumor therapy.

**Table 1:** Summary table of the effects of different stiffness models on cell function indicators.

Reference	Stiffness range and model	Outcome indicator
Evans ND et al (18)	0.041 MPa to 2.7 MPa polydimethylsiloxane	Stiffness promotes cell spreading, cell proliferation, mesendodermal gene expression and terminal osteogenic differentiation of ESCs
Yue M et al (20)	9, 25 and 42 kPa GelMA	Increased osteogenesis in a stiff and enhanced adipogenic/chondrogenic differentiation in a soft
Das RK et al (21)	0.2- 0.4 kPa fully synthetic polyisocyanopeptide	Stiffening as an important parameter that governs stem cell switched from adipogenesis to osteogenesis
Na J et al (22)	4.47 and 40kPa Polyacrylamide Gels	Stiff ECM promotes glycolysis, oxidative phosphorylation, and enhances antioxidant defense system during osteogenic differentiation in MSCs
Yeung T et al (28)	2- 55,000 Pa Polyacrylamide Gels	No actin stress fibers are seen in fibroblasts on soft surfaces, an abrupt change in spread area that occurs at 3000pa range
Wang W et al (29)	3 and 30 kpa Polyacrylamide Gels	Fibroblasts respond to substrate rigidity by recruiting more force-bearing integrins and modulating integrin sampling frequency of the ECM
Prager-Khoutorsky M et al (30)	4 kPa- 2Mpa Polyacrylamide Gel	On rigid surfaces, large and uniformly oriented focal adhesions are formed, whereas cells plated on compliant substrates form numerous small and radially oriented adhesions
Lee HH et al (31)	2.5, 9.0 and 21.5kPa Polyacrylamide Gels	Shp2 plays an essential role in the response of fibroblasts to matrix rigidity, on rigid surfaces, large focal adhesions and anisotropically oriented stress fibers are formed, whereas cells plated on compliant substrates form numerous small FAs
Plotnikov SV et al (32)	8.6–32 kPa Polyacrylamide Gels	Strengthening the molecular clutch via the FAK/phosphopaxillin/vinculin pathway broadens the range of rigidities over which dynamic ECM-rigidity sampling operates
Al-Hilal TA et al (33)	4 and 40 kPa Polyacrylamide Gels	Durotaxis drives fibroblast recruitment and activation via FAK–paxillin–YAP signalling
Mkrtschjan MA et al (39)	10, 100 and 400 kPa Polyacrylamide Gels	PIP2 is regulated by microtopography cues to mediate changes in collective migration velocity and lamellar architecture of fibroblasts
Asano S et al (42)	1–50 kPa Polyacrylamide Gels	Expression of α-SMA proteins, PDGF-induced chemotaxis and random walk migration of fibroblasts precultured on stiff substrateswas higher
Pineda-Hernandez A et al (43)	Helical, non-helical hydrogel	Stiffer hydrogels crosslinked with helical peptoids promoted higher proliferation rates and increased YAP nuclear localization
Monaghan-Benson E et al (44)	1 and 50kPa collagen-coated Gels	Enhanced matrix stiffness activates a RasGRF1/Ras signaling cascade that regulates the activity of AKT and ERK-dependent FOXO3a and Bim expression to alter cell survival
Ebrahimighaei R et al (42)	0.5, 8.0 and 50 kPa polyacrylamide Gels	Stiffness induced activation of YAP enhances the transcriptional activity of both TEAD and RUNX2 transcription factors
Woods K et al (46)	200, 300 and 1100 Pa mTG Gels	Cell proliferation, vimentin and TGF-β expression were increased in HMFs encapsulated in moderate and stiff hydrogels
Xu Z et al (49)	450–850 Pa Col I substrates	Soft substrates may promote neural reprogramming by inhibiting miR-615-3p targeting ITGB4
El-Mohri H et al (50)	1.3 kPa to 23 kPa GelMA	Increase in matrix stiffness resulted in enhanced fibroblast proliferation and stress fiber formation
Lerche M et al (59)	2.0 kPa collagen I coated Gels	Integrin α11β1 is essential in the integrin-collagen I binding dynamics and spreading of mammary gland stromal fibroblasts on soft collagen-I-coated substrates
Hui E et al (61)	1 and 15 kPa HA Gels	Integrin αvβ3 promoted increased spreading, actin stress fiber organization, and focal adhesion maturation on stiff elastic hydrogels, α5β1 binding suppressed these metrics
Balcioglu HE et al (62)	12 and 47 kPa fibronectin-stamped elastomeric pillars	Vinculin recruitment indicates a stiffness-dependent switch in vinculin function in cell-matrix adhesions
Schiller HB et al (64)	1.4, 10 and 35 kPa micropatterned polyacrylamide Gels	Integrin α5β1 accomplish force generation, whereas αv-class integrins mediate the structural adaptations to forces, which cooperatively enable cells to sense the rigidity of fibronectin-based microenvironments
Elosegui-Artola A et al (66)	5–29 kPa polyacrylamide Gels	Below 10–15 kPa, integrins unbind and release force before talin can unfold. Above the threshold, talin unfolds and binds to vinculin, leading to adhesion growth and YAP nuclear translocation
Li M et al (67)	6, 10, and 16 kPa FN‐coated substrate Gels	Integrin β1/Piezo1 activation/Ca2+ influx/HIF‐1α ubiquitination/VEGF, CXCL16 and IGFBP2 pathway participates in matrix stiffness‐driven HCC angiogenesis
Baghdadi MB et al (68)	1.5–30 kPa polyacrylamide Gels	PIEZO activation by niche stiffness intracellular Ca2+ influx is generated, subsequently repressing NOTCH pathway to induce secretory cell differentiation and modulating WNT signaling to maintain appropriate balance between self-renewal and proliferation
He J et al (69)	2 and 50 kPa collagen-coated polyacrylamide Gels	Increased matrix stiffness promotes skin fibrosis through the inflammatory Piezo1-Wnt2/Wnt11-CCL24 pathway. In turn, a stiffer skin microenvironment increases Piezo1 expression to exacerbate skin fibrosis aggression
Emig R et al (70)	2.7- 4.6 kPa CyPhyGels	Piezo1 Channels Contribute to the Regulation of Human Atrial Fibroblast Mechanical Properties and Matrix Stiffness Sensing
Batan D et al (76)	1–6 and 15–30 kPa PEG gels	YAP as a downstream target for TRPV4 activity on stiff microenvironments
Sharma S et al (77)	0.5 and 8 kPa hydrogels	TRPV4-elicited Ca2+ influx and myofibroblast differentiation are sensitized through interactions of cells with a matrix
Rahaman SO et al (78)	1, 8, and 25 kPa polyacrylamide Gels	TRPV4 channel activity was elevated when cells were plated on matrices of increasing stiffness, and matrix stiffness-dependent myofibroblast differentiation was reduced in response to TRVP4 inhibition.
McGowan et al (81)	0.08 and 100 kPa collagen Gels	Durotaxis is impaired in DDR2 deletion
Liu J et al (83)	2.6, 20, and 40 kPa polyacrylamide Gels	DDR1 underwent stiffness/collagen binding-stimulated liquid-liquid phase separation and co-condensed with LATS1 to inactivate LATS1
Dupont S et al (89)	0.7 and 40 kPa acrylamide Gels	YAP/TAZ are functionally required for differentiationnd and survival induced by ECM stiffness
Niu L et al (90)	4‐ 41 kPa gelatin Gels	Stiff matrix-induced high expression and nuclear localization of YAP in CFs, accompanied by enhanced cell activation
Liu F et al (91)	0.1–25 kpa Polyacrylamide Gels	Both YAP and TAZ accumulate in the nuclei of fibroblasts grown on pathologically stiff matrices but not physiologically compliant matrices
Codelia VA et al (94)	1 and 40 kpa polyacrylamide Gels	RAP2 is activated by low ECM stiffness, and RAP2 deletion blocks YAP/TAZ regulation by stiffness signals and promotes aberrant cell growth
Aragona M et al (95)	1.2 mg/ml and 3 mg/ml mixture of Growth Factor Matrigel and Collagen I	Mechanical forces are overarching regulators of YAP/TAZ setting responsiveness to Hippo, WNT, and GPCR signaling
Southern BD et al (100)	1, 8.0, 25 kPa Polyacrylamide Gels	Non-muscle myosin II activities fibroblast motility and polarization and promotes myofibroblast differentiation
Mittal N et al (102)	0.6, 1.3, 2.7, 6 and 12.7 kpa silicone Gels	Arp2/3 and formin enhance stiffness sensitivity by mechanically reinforcing the F-actin network
Noskovicova N et al (107)	2.3 and 2151 kPa silicone implants	Stiff silicones coated with a soft silicone layer reduced collagen deposition as well as myofibroblast activation
Szeto SG et al (113)	2kPa and 100kPa polyacrylamide Gels	Fibroblast growth on a soft matrix led to YAP/TAZ sequestration in the cytosol and impaired TGF-β-induced Smad2/3 nuclear accumulation and transcriptional activity
Piao J et al (117)	1, 10, 20 and 30 kpa acrylamide Gels	Stiffness could regulate EMT partially through miR-106b and its downstream target DAB2
Schrader J et al (118)	1 and 12 kpa Polyacrylamide gels	HCC proliferative index was higher, when the cells were cultured on stiff supports
McKenzie AJ et al (119)	3, 25 and 125 kpa polyacrylamide Gels	Cell spreading, focal adhesion formation, myosin light chain phosphorylation, and cellular traction forces all increase on stiffer matrices
Gamradt Pet al (129)	Pancreatic ductal adenocarcinoma	Stiffness-induced cancer-associated fibroblasts are responsible for immunosuppression in a platelet-derived growth factor ligand-dependent manner and inhibit CD8+ T-cell responses

In terms of research technologies, traditional methods primarily simulate ECM with different stiffness by adjusting gel polymerization degrees to investigate the effects of matrix stiffness on cellular behaviors. In recent years, atomic force microscopy (AFM), as a high-precision tool, can quantify key mechanical parameters such as cellular elastic modulus and hardness by measuring interactions between cells and microscale force-sensitive probes. Optical tweezers technology enables precise capture and manipulation of cells, applying microscale forces to measure cellular responses—facilitating both the study of cellular mechanical properties and the observation of cellular behaviors under mechanical stimulation. Furthermore, the development of single-cell multi-omics and spatial genomics technologies has not only refined the definition of fibroblast subsets but also deepened the understanding of their biological characteristics, providing critical insights into the diverse functional states of fibroblasts in response to physiological or disease-related triggers.

Epigenetic editing and single-cell CRISPRi have been used to screen out a class of cis-regulatory elements called “mechanical enhancers” that respond to changes in stiffness (130). Epigenome editing of mechanical enhancers will be a powerful tool to precisely regulate the response of cells to the mechanical microenvironment, and has broad application prospects in cell engineering and gene therapy. It can regulate the positive feedback loop of pathogenicity while maintaining important signaling functions. Avoiding strongly regulated genes at the same time will disrupt both pathogenic and essential functions, and may lead to new treatment strategies for mechano-sensitive diseases. Following the development of scRNA-seq (131), researchers have identified different subtypes of fibroblast, which provides opportunities for precise inhibition of fibrogenic fibroblast. Hence, by learning the track of (myo)fibroblast in the tissue fibrosis, we may be able to detect a potential therapeutic target that does not affect the function of normal fibroblast. Future research should focus on three core directions:First, deepening the molecular mechanisms of mechanotransduction—with emphasis on exploring the regulatory networks underlying multi-sensor synergistic perception, signal cross-integration, and mechanical memory formation, while clarifying the interaction rules between mechanical and biochemical signals. Second, strengthening disease-specific research—analyzing the spatiotemporal characteristics of abnormal ECM stiffness in different disease types, screening for disease-specific mechanical biomarkers, and providing new indicators for early disease diagnosis.Third, advancing translational medicine research—developing specific drugs targeting mechanotransduction pathways and stiffness-sensitive drug delivery systems based on mechanistic insights, or reversing abnormal cellular phenotypes by regulating ECM stiffness, thereby offering novel therapeutic strategies for diseases such as fibrosis and tumors.

## 5 Conclusion

In summary, the regulation of fibroblast functions by ECM stiffness constitutes a multi-layered, modular, and highly integrated signal regulatory system. It closely links macroscopic physical forces to microscopic molecular events and gene expression regulation, not only deepening the fundamental understanding of cell-microenvironment interactions but also building a bridge for the interdisciplinary integration of physics/mechanics and life sciences. The findings from this research not only provide a theoretical basis for the precise design of tissue engineering scaffolds in regenerative medicine but also hold promise for breaking through the limitations of traditional biochemical interventions, opening up a new field of “mechanical regulatory therapy”. With further advancements, targeted regulation of the mechanical microenvironment is expected to enable precise intervention in disease progression, providing novel theoretical foundations and strategic directions for regenerative medicine, tissue engineering, cancer therapy, and anti-fibrotic drug development.
